# First-ever Ischemic Stroke after a Flight in a Patient with Prior Poliomyelitis

**DOI:** 10.4137/cpath.s476

**Published:** 2008-02-01

**Authors:** Cheng-Chiang Chang, Shin-Tsu Chang, Chih-Hung Ku, Shang-Lin Chiang, Hsiao-Ying Chang, Min-Hsin Lai, Kao-Chung Tsai, Liang-Cheng Chen

**Affiliations:** 1 Department of Physical Medicine and Rehabilitation, Tri-Service General Hospital, School of Medicine, National Defense Medical Center, Taipei, Taiwan; 2 School of Public Health, National Defense Medical Center, Taipei. Taiwan; 3 Department of Nursing, Tri-Service General Hospital, Taipei, Taiwan

**Keywords:** poliomyelitis, ischemic stroke, anticardiolipin antibodies, dehydration, cabin pressurization, decompression sickness

## Abstract

Survivors of poliomyelitis sometimes travel by air with mobility assistance. However, prolonged seating during long-haul flights may also possibly produce stroke events on polio-inflicted patients. A 48-year-old polio-inflicted male suffered a stroke after an extended flight. A two-dimensional echocardiography was normal without detected patent foramen ovale or dyskinetic segment. The venodynamic variables were all within normal limits. MR Imaging studies revealed acute cerebral infarction in the distribution of the right middle cerebral artery and posterior watershed area. Hematological examination revealed positive anti-cardiolipin IgG antibody which might contribute to the risk of thrombosis as an underlying condition in addition to immobilization. This is the first presentation of ischemic stroke after a flight in a patient with prior poliomyelitis. In addition to decompression sickness, economy class stroke syndrome and postpoliomyelitis syndrome, the physician should also take other coagulation disorders into consideration during the investigation.

## Introduction

Poliomyelitis, first described in 1789, originates with poliovirus infection belonging to a member of a group of viruses designated as enteroviruses (Howard, 2005). Less than 1% of patients infected with the poliovirus develop flaccid paralysis. Paralysis appears anywhere within a few days after symptoms including weakness or paralysis of the limbs, and, possibly, the muscles controlling speech, swallowing, and breathing (Howard, 2005). Survivors of poliomyelitis will sometimes experience air travel with mobility aids. However, prolonged seating during long-haul flights may also possibly produce a stroke event on polio-inflicted patients.

When it comes to aviation, cabin pressurization is possibly one of the most outstanding and assumptive progress in allowing extended flights at high altitudes and diminishing the risk of altitude-related disease (Stuart et al. 1995). However, cabin pressurization does hold its limitations. While the existence of anti-cardiolipin antibodies might contribute to the risk of thrombosis as an underlying condition, they have been linked to the identification of patients subject to recurrent arterial and/or venous thrombosis which is also supposed to be triggered by dehydration induced by cabin pressurization during prolonged flight in addition to immobilization (Schreijer et al. 2006). Reduction of diminished humidity is especially important on extended flights because of the air-drying effect on the exposed mucus membranes and the potential for dehydration possibly leading to ischemic stroke especially in patients with prior poliomyelitis.

## Case Report

A 48-year-old polio-inflicted male was admitted to the hospital because of the sudden onset of slurred speech and left hemiparesis. Although he had suffered from poliomyelitis causing weakness of the left arm and right leg at the age of 1 year, he had experienced no weakness for several decades since complete recovery of poliomyelitis. He had used calipers (leg braces) as a child and young adult for assistive mobility, but had walked well. The patient, a non-smoker, gave a history of selling gum to earn money for several decades. For the first-ever chance to go abroad by airplane, he felt nervous and uneasy and thus drank no more than a glass of water during the flight. General weakness of four limbs and trunk muscles suddenly developed six hours after getting off the plane, even difficulty in turning over on the bed. He did not pay much attention to it and restored his locomotion ability with improvement of the muscle strength on the next day. During his two-week period stay in a tropical country, with humidity ranging from 69%–95%, he had no significant medical complaints. One hour after coming back to his own country and taking a shower, he fell down on the floor in his bedroom suddenly with difficulty in getting up from the floor for two hours. However, he did not go to see the doctor and the symptoms resolved spontaneously. Unfortunately, one night after several weeks, he experienced episodic loss of consciousness and was sent to our ER on the next day.

On initial physical examination, his vital parameters were normal. His pulse was 80 beats/minute and the blood-pressure reading taken from the right brachial artery in supine position was 154/90 mm of Hg. All the peripheral pulses including the carotids were well felt. On neurological examination, he was conscious, cooperative, well oriented in time, space and person but showed moderate hyposthenia and hypoaesthesia, impaired muscle power and increase of deep tendon reflexes on the left side, inferior left VII cranial nerve palsy and mild dysarthria without gaze or visual field deficit. There were no cerebella signs. Examination of the other systems was unremarkable.

**Figure 1.**
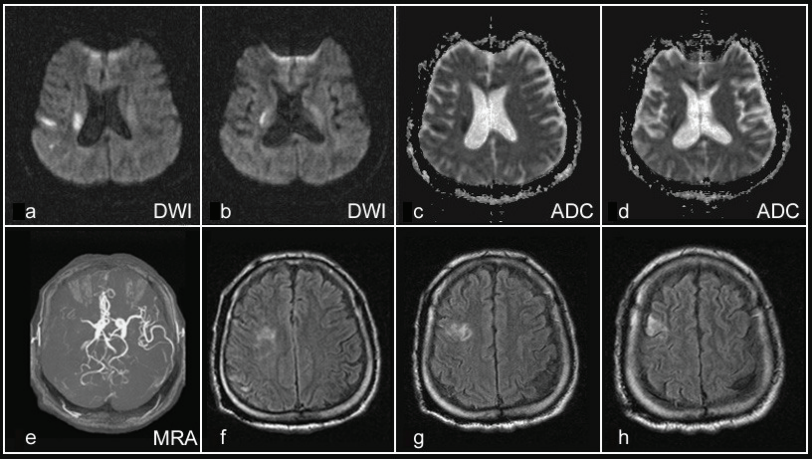
Imaging findings of the patient. Axial diffusion-weighted imaging (DWI) of brain MRI reveals several small acute infarcts involving the frontal and parietal lobes, right corona radiata and dorsal putamen shown with bright signal in the distribution of the right MCA territory and in the territories of the posterior watershed area compatible with the area of the apparent diffusion coefficient (ADC) reduction (**a–d**). The 3D time-offlight (TOF) MR angiography demonstrates abrupt disruption in mid-segment of M1 of right MCA (**e**). Long TR sequences demonstrate high signal abnormality centered in the right temporal, occipital, and middle frontal lobes suggesting old ischemia or infarct on the right cerebral hemisphere (**f–h**).

On investigations his hemogram, routine urine, and stool examination were normal. Measurement of anticardiolipin (aCL) antibodies (Abs) revealed negative results for IgA and IgM aCL Abs with the exception of IgG aCL Ab (24 GPL/mL; normal range <13 GPL/mL). His ECG revealed evidence of sinus tachycardia. A two-dimensional echocardiography was normal without detected patent foramen ovale or dyskinetic segment. Transfer Function Index (TFI) or Pulsatility Index Ratio (PIR) screening was normal. The venodynamic variables measured including segmental venous capacitance (SVC), maximum venous outflow (MVO), and venous emptying time (VET) at the calf, ankle and great-toe were all within normal limits. Carotid duplex scanning demonstrated intact common carotid, internal carotid and external carotid arteries. Radiograph of his chest revealed marked scoliosis of the thoracolumbar spine, chest deformity and elevated right diaphragm. MR Imaging studies (Fig. 1) revealed an acute cerebral infarction in the distribution of the right middle cerebral artery and in the territories of the posterior watershed area (area between right MCA and right PCA). Mildly atrophic change of the right cerebral hemisphere with multiple small areas of old cerebral infarction at the frontal, medial temporal and occipital lobes was noted. A diagnosis of cerebral infarction of right MCA territory with left hemiparesis was made. The patient was administered with pentoxifylline and antiplatelet agents immediately after the diagnosis was confirmed. Eventually, he was treated by comprehensive physiotherapy with progressive improvement for the past one year.

## Discussion

Activation of coagulation system during air travel has been a popular issue recently, and most of the researches attempt to provide further insight into discovering whether flying leads to a hypercoagulable state (Schreijer et al. 2006). One recent study published by Schreijer et al. in Lancet demonstrated more coagulation abnormalities after a flight than after movie-watching or normal daily activity, and the authors demonstrated that flight can create a pathophysiology prone to thrombus formation in addition to immobilization alone (Schreijer et al. 2006).

Venous thromboembolism associated with travelling, or economic class stroke syndrome, has been more and more emphasized. In our case, dehydration induced by cabin pressurization during prolonged flight in post-polio status would have been the trigger of thrombosis, while the existence of aCL Ab might contribute to the risk of thrombosis as an underlying condition. Besides, the economy class stroke syndrome or Traveler’s thrombosis (deep vein thrombosis), a thrombosis that occurs in the deep venous system of the lower extremities (with or without pulmonary embolism complications) of a person, who did not show any signs of acute venous embolism at the time of departure, resulting from a prolonged seating during flights might also taken in to consideration for the CVA event. The low mobility fostered by long-haul flights favors the developments of deep vein thrombosis in patients with existing risk factors including: pressure on the upper thighs, caused by prolonged sitting and low mobility in narrow seats and rows; low air humidity on board can favor the formation of blood clots in cases where passenger may be lacking fluids. It has been reported to be associated with the presence of a patent foramen ovale (PFO). Patients with PFO may be vulnerable to stroke from paradoxic embolism (Kakkos and Geroulakos, 2004). Kakkos et al. reported a patient with economy class stroke syndrome due to an PFO with right to left shunt demonstrated by transoesophageal contrast echocardiogram (Kakkos and Geroulakos, 2004), whereas there was no patent foramen ovale or dyskinetic segment detected in our patient under the similar circumstances after a prolonged fight. Still, the correlation between the economy class stroke syndrome and aCL Abs should be further investigated to clarify whether aCL Abs would exacerbate the economy class stroke syndrome or not.

Anticardiolipin antibodies (particular high-titer IgG) which are associated with clotting, fetal loss, thrombocytopenia, and valvular heart disease, have also been linked to the identification of patients subject to recurrent arterial and/or venous thrombosis. Without having systemic lupus erythematosus, patients were classified to primary antiphospholipid syndrome for non-lupus patients having anti-cardiolipin antibodies (Hughes, 1984). However, there has been no linkage between the incidence of anti-cardiolipin antibodies and post-polio status in the literature.

Patients with large-artery atherosclerotic disease and stroke of unknown etiology had higher frequencies of increased aCL Ab than those in control subjects. Demonstrated to be independent stroke risk factors across the 3 ethnic groups studied, conferring a 4-fold increased risk of ischemic stroke, aCL-IgG selectively increases in patients with large-artery atherosclerosis and stroke of unknown etiology, reflecting selective activation of humoral immunity for aCL in the pathogenesis of cerebral ischemia (Chen et al. 1999; Tuhrim et al. 1999).

This represents the first report of first-ever ischemic stroke in a patient with prior poliomyelitis as the result of environmental triggers. The concurrent elevation of aCL Ab and dehydration induced by cabin pressurization suggests that aCL Ab and their proposed thrombogenic and vascular injury consequences, might contribute to development of stroke in patients with prior poliomyelitis. Carhuapoma et al. reported that patients with positive aCL concomitant with central venous thrombosis were associated with dehydration. In addition, aCL may be an important factor contributing to development of central vein thrombosis (CVT) even in the presence of other potential etiologies or risk factors. Onset of aCL-positive CVT occurs at a relative young age and with relatively more extensive superficial and deep cerebral venous system involvement than aCL-negative CVT (Carhuapoma et al. 1997). Because of the scarcity of the reported cases, whether the presence or absence of aCL in patients with CVT has clinical relevance remains unknown. Furthermore, the exact mechanism by which aCL promotes thrombosis and the therapy of choice also remain largely unknown. The etiology of thrombosis in patients with aCL Ab still has been the object of recent investigations (Chen et al. 1999; Tuhrim et al. 1999).

Cabin pressurization does hold its limitations and problems (Stuart et al. 1995). Most jet aircraft pressurization equipment applies bleed air from jet engines. Bleed air is very hot and requires thorough cooling before it can be released into the cabin. The basic composition of the air is essentially the same as sea-level air, but due to the high temperature of the air when it enters the pressurization system from the turbines, it is excessively dry. This diminished humidity is especially important on extended flights because of the air-drying effect on the exposed mucus membranes and the potential for dehydration (Stuart et al. 1995).

In addition to the aforementioned dryness of the air, the air in the aircraft at the high altitude is usually not pressurized to the same atmosphere at sea-level. Typical pressure in the aircraft at the high altitude still ranges from 5000 to 8000 feet above sea level, which is maintained despite the altitude the aircraft reaches (Rios-Tejada et al. 1997). Therefore, we should never neglect the similar conditions that can be deteriorated by this inevitable change, such as decompression sickness (DCS) because of the previous case reports of neurological manifestation of arterial gas embolism following standard altitude chamber flight at flight level 28,000 feet (Rios-Tejada et al. 1997). Cerebral involvement in DCS has been investigated recently in radiology literature. Levine et al. reported that MRI within two weeks after DCS in two divers revealed subcortical white matter lesions (Levine, 1989). Aksoy stated that MR imaging after the injury demonstrated high signal intensities in subcortical white matter might reflect the residues of subclinical cerebral DCS injury (Aksoy, 2003). We excluded the possibility of DCS by the MR imaging pattern of multifocal acute infarcts in our case predominantly involving the frontal and parietal lobes, right corona radiata and dorsal putamen distributed chiefly at the right MCA and watershed area of right MCA+PCA instead of mainly subcortical white matter in the previous literature (Aksoy, 2003).

On the other hand, postpoliomyelitis syndrome (PPS) should be taken into consideration because the symptoms are similar to those of stroke. PPS is defined as a syndrome characterized by the delayed appearance of new neuromuscular symptoms many years later in patients with a prior history of symptomatic poliomyelitis (Jubelt and Agre, 2000). It is clinically manifestated with new muscular weakness and atrophy of the limbs, bul-bar innervated musculature, and muscles of respiration, usually combined with excessive fatigue, joint pain, and reduced stamina (Jubelt and Agre, 2000). A thorough history taking, physical examination, blood analysis and imaging study should be performed to exclude the possibility of PPS. Additionally, with the help of MRI of brain, our patient was provided a well-being care as a result of avoidance of developing further deterioration if being misdiagnosed as PPS.

**Figure 2.**
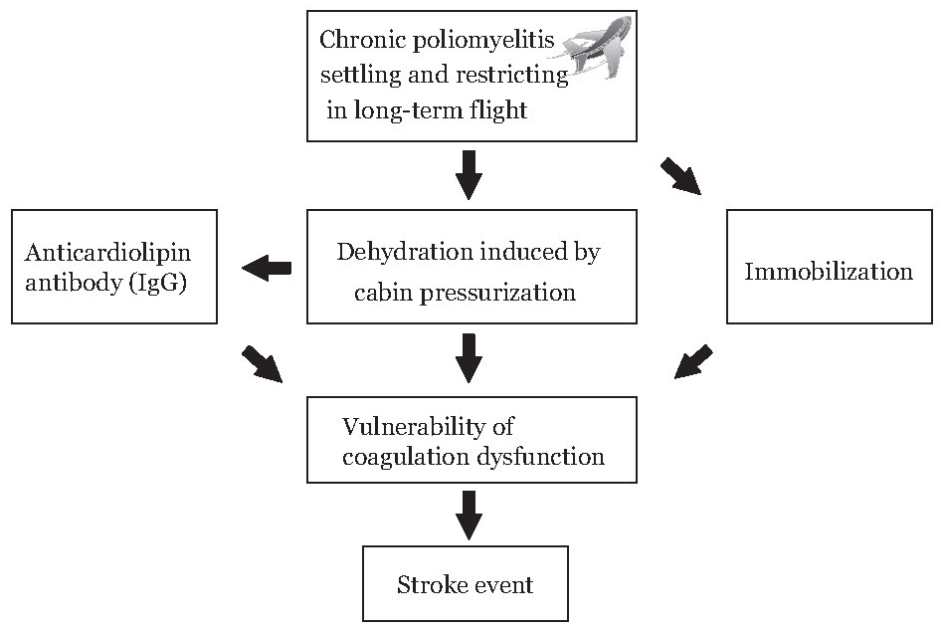
Proposed pathophysiology of the stroke attack after prolonged flight in our case who was the patient of chronic poliomyelitis.

In conclusion, in addition to decompression sickness, economy class stroke syndrome and postpoliomyelitis syndrome, the physician should also take other coagulation disorders into consideration during the investigation. Economy class stroke syndrome and their potential association with antiphospholipid antibodies should be further investigated in addition to the previous causes including PFO in the patients with economy class stroke syndrome. However, reduction of diminished humidity is especially important on extended flights because of the air-drying effect resulting in possible dehydration leading to ischemic stroke, especially among those having been tested positive for anti-cardiolipin IgG antibody in patients with prior poliomyelitis while prolonged immobilization may not be the only mechanism involved in the activation of coagulation system during air travel (Fig. 2).
